# Cognitive Behavior Evaluation Based on Physiological Parameters among Young Healthy Subjects with Yoga as Intervention

**DOI:** 10.1155/2015/821061

**Published:** 2015-02-11

**Authors:** H. Nagendra, Vinod Kumar, S. Mukherjee

**Affiliations:** ^1^Faculty in E & CE Department, Poojya Doddappa Appa College of Engineering, Kalaburagi 585 102, India; ^2^Department of Electrical Engineering, Indian Institute of Technology Roorkee, Roorkee 247 667, India; ^3^Moradabad Institute of Technology, Moradabad 244 001, India

## Abstract

*Objective*. To investigate the effect of yoga practice on cognitive skills, autonomic nervous system, and heart rate variability by analyzing physiological parameters.* Methods*. The study was conducted on 30 normal young healthy engineering students. They were randomly selected into two groups: yoga group and control group. The yoga group practiced yoga one and half hour per day for six days in a week, for a period of five months.* Results*. The yoga practising group showed increased *α*, *β*, and *δ* EEG band powers and significant reduction in *θ* and *γ* band powers. The increased *α* and *β* power can represent enhanced cognitive functions such as memory and concentration, and that of *δ* signifies synchronization of brain activity. The heart rate index *θ*/*α* decreased, neural activity *β*/*θ* increased, attention resource index *β*/(*α* + *θ*) increased, executive load index (*δ* + *θ*)/*α* decreased, and the ratio (*δ* + *θ*)/(*α* + *β*) decreased. The yoga practice group showed improvement in heart rate variability, increased SDNN/RMSSD, and reduction in LF/HF ratio.* Conclusion*. Yoga practising group showed significant improvement in various cognitive functions, such as performance enhancement, neural activity, attention, and executive function. It also resulted in increase in the heart rate variability, parasympathetic nervous system activity, and balanced autonomic nervous system reactivity.

## 1. Introduction

The practice of yoga synchronizes human physiology through controlled postures, breathing, meditation, a set of regular physical exercises, and relaxations [1–4]. Certain types of yoga practice improve autonomic nervous system by modulating parasympathetic and sympathetic activity, significant changes in brain rhythms, sensory motor rhythm, regulation of breathing rate, and improvement in the cardiac activity and enhance the sense of “well-being” [5, 6]. Yoga practice has many physiological benefits including increase of heart rate variability (HRV), decreased blood pressure, and increase in respiratory rate and baroreflex sensitivity and balances autonomic nervous system (ANS) activity by reducing sympathetic activity and increasing parasympathetic activity [2]. Previous research suggests that yoga practices have immense impact on performance of central nervous system and improve their attention, concentration, and other cognitive faculties [7]. Regular practice of yoga has benefits in the improvement of the body, mind, and spirit, guiding to a healthier and more fulfilling life [8]. The practice of yoga can increase grey matter volumes in temporal and frontal lobes, producing positive impacts on mental health and improved cognitive functions [3]. Study also suggested that yoga practice could also bring improvement in tasks which are related to selective attention, concentration, visual processing capacity, and enhancement in motor activity [9]. In another study, the practice of yoga resulted in improved eye-hand coordination, improved reversal skills, speed, accuracy, and enhanced cognitive processes [3]. Practicing of pranayama, asanas, and meditation resulted in improved verbal skills, improvement in hand-eye coordination, and improved neural performances [3, 10]. It is believed that the practice of yoga can also result in changes in perception, attention, and cognition. Investigations have shown the beneficial effects of yoga on cognition, such as increased performances on visual and verbal memory and improved memory scores [11].

Compared to physical exercise yoga may be more effective or even better in improving health related conditions. Despite corpus of research on the subjects, the lack of evidence based on scientific approaches has limited the application of yoga as an accepted method for improvement of health [11]. Hence further research is needed on the impact of yoga and its potential benefits on healthy subjects. Thus yoga offers many positive effects on cognitive faculties, reduction of stress, and emotional intensity. Previous studies were mainly conducted on unhealthy or relatively elder subjects. The focus was generally on physical and neurological benefits. Further investigation is required to study the potential benefits of yoga on cognitive functions and their relation with physiological parameters. In this study the effects of yoga practices on cognitive skills, autonomic nervous system, heart rate variability, and mental health are analyzed in terms of physiological parameters such as electroencephalogram and electrocardiogram.

Therefore the objective of this study is to investigate the effectiveness of yoga practice and to evaluate physiological parameters related to cognitive aspects on novice subjects. The study primarily focused the effect of yoga on cognitive behavior in terms of physiological parameters. In the current study, the yoga practice involved combined practice of easy asanas (postures), meditation, and pranayama (breathing exercise). It is known that yoga involving relaxation techniques improve the functioning of cardiovascular autonomic nervous system. Yoga is correlated with decreased sympathetic adrenergic receptor sensitivity, which might affect cardiovascular response during stress [12].

### 1.1. Heart Rate Variability and Its Indices

Heart rate variability (HRV) is a measure of deviations in the interbeat R-R intervals. It is a noninvasive method used to assess the functioning of the autonomic nervous system (ANS), which is responsible for the regulations of many physiological processes of the human being [13]. The HRV is caused due to changes in input to the sinus node from the autonomic nervous system (ANS) [14]. The sinus node (natural pace maker) is one of the major components of the cardiac conduction system that regulates the heart rate (HR) by controlling sympathetic nervous system (SNS) and parasympathetic nervous system (PNS) limbs of the ANS [15]. Higher HRV is an indicator of adequate adaptation to the new environment and effective functioning of the ANS, while lower HRV is an indicator of inadequate adaptation of ANS and poor physiological function of the individuals [13]. HRV and HR are inversely correlated. The escalation in the HR is due to increased sympathetic and decreased parasympathetic activity, whereas its reduction mainly depends on the dominance of parasympathetic activity.

Generally, for HRV analysis, parameters can be computed by two methods [13, 15–17].

- Time domain measures are directly computed from the time series of the RR intervals. In the literature there are many time domain measures available for HRV analysis. In this paper the following indices are used for its analysis:
- mHR: mean RR intervals;
- mHRV: mean heart rate variability and it indicates the total amount of deviations of both instantaneous HR and RR intervals. It reflects sympathetic and parasympathetic activity of the ANS on the sinus node of the heart;
- SDNN: standard deviation of all NN intervals and an indicative of global HRV. It indicates all the long term elements and circadian rhythms responsible for variability in the recording interval;
- RMSSD: the Square roots of the mean of the sum of the squares of differences between adjacent NN intervals and it reflects the short cyclical variability in the autonomic tone that is largely vagally mediated;
- CVRR: coefficient of variations of RR intervals and it is used to reflect the parasympathetic nervous system activity;
- the important time domain parameters are shown in Table 1.
- Frequency domain parameters are computed by applying fast Fourier transform (FFT) to the time series of the raw RR intervals. FFT is the most powerful and efficient algorithm used to break the HRV signal into a series of sine and cosine components. This Fourier transformed signal is further translated to power spectrum by squaring magnitude of each [18]. The fundamental frequency components were computed by integrating the periodogram. Generally, the power spectrum can be classified into the following four groups [19].
- Very low frequency (VLF: 0.0033–0.04 Hz) power: the function of this frequency range is not well defined but sometimes it can be used as the index of sympathetic activity of ANS.
- Low frequency (LF: 0.04–0.15 Hz) power: this band is complex in nature and an index of both sympathetic and parasympathetic activity and influences HRV patterns.
- High frequency (HF: 0.15–0.4 Hz) power: it is the index of parasympathetic activity and is used to indicate slow changes in the HR.
- Very high frequency (VHF: >0.4 Hz): this frequency is generally considered as noise and has no clinical significance.
- LF/HF ratio: It reflects the overall balance of the ANS. The lower ratio is recommended by the task force. In normal, in resting condition, this ratio lies in the range of 1 and 2.
- Total power (TP): variance of all NN intervals in the frequency range less than 0.4 Hz.

The VLF, LF, HF, and TP are expressed in ms^2^ units, when computed in absolute values. The important frequency domain parameters used for the computation are shown in Table 2.

The spectral parameters of HRV are usually normalized to minimize the effect of redundancy inherent in them in most of the research work. The important frequency domain parameters are shown in Figure 2.

### 1.2. EEG Band Frequencies and Cognitive Processes

The brain activity which changes continuously with time is called “electroencephalogram” (EEG), which can be used to investigate the cognitive abilities and memory executions of individuals in terms of its band of frequencies.

The EEG is highly complex and is combination of five different frequency waveforms, namely, *δ* (delta), *θ* (theta), *β* (beta), *α* (alpha), and *γ* (gamma) waves, respectively [20]. The amplitude of the brain waves is approximately in the range of 10 *μ*V to 250 *μ*V and the frequency varies between 0.5 Hz and 100 Hz. The frequency range and their characteristics are shown in Table 3.

The EEG waveforms may be global or localized to the specific areas on the scalp. This kind of electrical data is important to study the correlation between yoga asanas and physiological states, because any shift in the EEG frequency range reflects the physiological arousal. The various EEG ratio indices and their physiological and cognitive interpretations are shown in Table 4.

### 1.3. Extraction of EEG Band Frequencies Using Discrete Wavelet Transforms (DWT)

Discrete wavelet transforms (DWT) are widely used for the analysis of physiological signals as compared to the classical techniques such as fast Fourier transforms (FFT). When FFT is applied on the time series signal, the signal information is available in the form of spectral parameters. That is, the whole time domain information will be lost. It is equivalent to windowed Fourier transform and can be used to measure both the time and frequency changes of a signal [21].

The DWT splits the input signal into approximation (trend) and detailed coefficients (fluctuation), respectively. The approximation coefficient can further be split into a new approximation and detailed coefficients. This process is continued progressively to get a new set of approximation and detailed coefficients of a signal at various levels of decomposition [22]. The selection of analyzing wavelet is called mother wavelet and number of decomposition levels to be carried out is the critical point. The mother wavelet determines the shape of the signal to be decomposed. In this paper the wavelet function db4 is used to extract five frequency bands (*δ*, *θ*, *α*, *β*, and *γ*) of EEG signal. The application of higher order wavelet function such as db20 produces large number of coefficients. Larger number of coefficients average out the detail components of the signal and fail to detect fast moving signals such as EEG. To retrieve the information at a specific instant of time, the wavelets with less number of coefficients are better choice. The lower order wavelet function db4 has good time and frequency localization properties, and in addition this wavelet has similar morphology as that of EEG signal to be detected. Therefore, db4 wavelets are better choice for precisely detecting fast moving transients and short duration information signals. Thus, by the process of decomposition, DWT can detect the important hidden features from the original signal.

In this study the EEG signal was acquired with sampling frequency of 500 Hz. The useful information of this signal lies in the range of 0.5–70 Hz. Hence a level of 7 using db4 was applied to decompose the EEG signal into its approximate (A1–A7) and detail (D1–D7) coefficients. After the seventh level of decomposition, the band of frequencies obtained are D1 (250–500 Hz), A1 (0–250 Hz), D2 (125–250 Hz), A2 (0–125 Hz), D3 (62.5–125 Hz), A3 (0–62.5 Hz), D4 (31.25–62.5 Hz), A4 (0–31.25 Hz), D5 (15.625–31.25 Hz), A5 (0–15.625 Hz), D6 (7.8125–15.625 Hz), A6 (0–7.8125 Hz), D7 (3.906–7.813 Hz), and A7 (0–3.906 Hz), respectively. The decomposition levels from D1 to D3 were considered as noise components and hence excluded from the analysis. The finer detailed coefficients from levels D4–D7 and final approximate coefficients from level A7 are retained as they approximately represent the EEG physiological frequency subbands of *γ*, *β*, *α*, *θ*, and *δ*, respectively. These five frequency bands are analyzed to investigate different cognitive effects due to yoga among healthy subjects. Different EEG ratios used in this study to investigate cognitive performances in terms of physiological parameters are shown in Figure 4, which are derived from various sources.

## 2. Methodology and Experimental Procedure

### 2.1. Subjects

The total number of subjects who participated in the experiment was 30 young healthy graduate and postgraduate engineering students of IIT Roorkee (male = 27, female = 3). All the subjects were right handed with normal eye sight. The study population was divided into two groups: experimental group and control group. In this study the sample size is relatively small and both groups have the same size. The study population was randomly assigned to either of the groups by block randomization method to achieve the balance. The block size of two was used. Both participants and investigators were unaware of the groups to be assigned in advance. Each group consisted of 15 subjects with two females in experimental group and one in control group. The mean and standard deviation of each group were 22.42 ± 2.30 and 23.67 ± 2.09, respectively. The same subjects were chosen for both experimental and control groups to diminish misperceiving influences and make the study more effective. Subjects with previous yoga practice, history of alcohol consumption, smoking, and any other drug consumption were excluded from this study. The participants were informed* a priori* about the study and their consent was obtained. Subjects participated voluntarily and cooperated throughout the training period. The subjects were asked to not to deviate their regular life style during this study. All the subjects of experimental group obtained same yoga training for a period of five months for 1.5 hours per day between 6 p.m and 7.30 p.m.

In this study the physiological parameters such as ECG and their ratio indices have been evaluated to assess the cognitive benefits of the yoga practice along with its well established health benefits. This may provide the window for further investigation to correlate the actual measures of cognitive functions and their physiological parameters.

The practice of yoga schedule consisted of prayer, pranayama (breathing techniques), and simple yogic postures. Explanations on stress management, importance of meditation, and yoga in everyday life were also briefed.

The subjects practiced yoga under observation of trained yoga instructor. During practice session various types of asanas (postural exercises), pranayama (breathing techniques), and* dhyana* (meditation) were performed. These asanas increase the strength, concentration, will power, and mindfulness by manipulating the natural energy of the body [1, 32, 33].

1. Standing asanas (postures): they consisted of surya namaskar, dandasana, urdhave asana, trikonasana, ardha asana, hasta padasana, mahavir asana, and vatayanasana.
2. Sitting asanas (postures): they include mandook asana and oorm asana, ushtra asan, ardha matsyendrasana, vakrasana, supt asana, matsyendrasana, uttan mandukasana, vakasana, mayoor asan, padm vak asan, padma mayurasana, pashchimottanasan, eka padangusthasana, vipreet pad asan, and purna chakrasana.
3. Asana (posture) lying on back: this includes uttan pad asana, pawanmuktasana, market asan, shreeshan, sarvangasana, halasana, setu bandhasana, and chakrasana.
4. Asana (posture) lying on stomach: this includes naukasana, yan asan, shalabh asan, and dhanurasana.
5. Pranayama (breathing) and kriya include Anulom-vilom, kapalbhati, Ujjayi pranayama, Bhramari pranayama, sheetali pranayama, sheetkari pranayama, surya bheda pranayama, bhastrika pranayama, bahya pranayama, udgeeth pranayama, kaki mudra, and shanmukhi mudra. Everyday practice session was concluded with prayer and meditation.

### 2.2. Recording ECG and EEG Signals and Analysis

Both ECG and EEG signals were recorded simultaneously using BIOPAC MP150 System (EEG100C = 10 nos and ECG100C = 3 nos) with Acqknowledge 4.0 software.

ECG signal was recorded using five electrodes by connecting to left and right wrinkles and left and right arms and one electrode at chest. Before fixing the electrodes they were cleaned and electrode gel was applied to reduce the skin resistance to get good quality of recording signal. EEG signal was recorded by fixing the CAP100C on the scalp of the subjects. This cap was made of Lycra type fabric with 20 reusable tin electrodes attached to it, according to the international 10–20 norms. Before fixing the cap on subjects scalp, the electrodes were cleaned with saline water and electrode gel was applied. This maintains the resistance below 5 kΩ between the scalp and electrodes.

All the data were collected during 6 p.m. to 7.30 p.m. at the yoga centre of the temple premises in two stages. The data collected at the beginning of the intervention period was considered as the first stage, during which five minutes of baseline ECG and EEG signals were recorded from subjects of both experimental and control groups in sitting position with eyes closed. The baseline signal was saved on the hard disk for offline processing.

Both ECG and EEG signals were recorded with a sampling frequency of 512 Hz to have better resolution of R-R time interval series and EEG activity.

The experimental group practiced yoga for a period of five months for 1.5 hr per day in the evening from 6 p.m. to 7.30 p.m. The control group was asked not to practice any form of yogic practices or physical exercises during this period. The end of five months yoga training period was considered as second stage. During this stage again both ECG and EEG data were collected from experimental and control group for a period of 10 minutes. During recording, subjects were asked to minimize eye blinks and avoid body movements to minimize any artifacts that could be introduced. If artifacts were introduced due to uncontrolled body movements or eye blinks or due to technical reasons, the recording time was prolonged for a few more minutes. The data was again saved on the hard disk for offline processing. These data were used for the evaluation of various cognitive functions in terms of physiological parameters.

Though maximum care was taken, the recorded data was contaminated with many artifacts. Manual editing was performed for both ECG and EEG signals. The RR intervals were then extracted from the Acqknowledge 4.0 software which uses modified Pan and Tompkins algorithm. The intervals less than 300 ms and above 1200 ms were eliminated from time series data set and were saved in text format for further processing using MATLAB 7.1. Any data whose standard deviation was less than or equal to three times the standard deviation was considered outliers and removed from the data before determining the time domain parameters of heart rate variability (HRV). The artifact free data was segmented into five groups with 10 seconds segments each. The average value of each 10 seconds data was used in the analysis. The important time domain measures of HRV such as mean HRV, SDANN, RMSSD, and mean HR were computed.

The frequency domain parameters, namely, VLF, LF, HF, LF : HF ratio, and VHF, were extracted from the FFT algorithm. The normalized values were computed by dividing the respective frequencies by total power minus VLF. The normalization reduces the effect of LF and HF on total power. In conformity with the task force recommendation, The artifact free signal of two minutes duration was used for computing frequency domain parameters. 

*Hypotheses of the Study*

*Hypothesis 1.* There is no significant difference in physiological parameters (HR, HRV) and cognitive functions of the subjects of experimental group before and after yoga training (intervention).

*Hypothesis 2.* There is no significant difference in physiological parameters (HR, HRV) and cognitive functions of the subjects of control group before and after yoga training (intervention).

## 3. Results and Discussion

Results are grouped in two parts: firstly, HRV analysis, which includes time and frequency domain parameters; secondly, cognitive performance evaluation based on EEG engagement indices. Both EEG and ECG signals reflect global arousal or alertness of the brain [34].

### 3.1. HRV Analysis

The heart rate variability (HRV) is an indicator of cardiac ANS and HR is controlled by neural activity [35]. The yogic exercise particularly pranayama (breathing techniques) activates ANS. The yoga practicing group showed significant increase in HRV (*P* < 0.0304) and reduction in resting HR (*P* < 0.0389). The significant reduction in resting HR indicates a relaxed state of physiology and increased mental alertness. Depending on left or right cerebral hemispherical dominance, there will be improvement in spatial or verbal skills. The SDNN which reflects the total power significantly increased (*P* < 0.0012). The RMSSD, an indicator of parasympathetic activity, also increased significantly (*P* < 0.0058). The ratio of SDNN/RMSSD which is surrogate of LF/HF ratio [36] increased significantly (*P* < 0.0039). LF was significantly decreased (*P* < 0.0002) while HF was increased (*P* < 0.0003). The decrease of LF and increase of HF reflects the improvement in the dominance in the parasympathetic activity. A significant improvement in HRV may be due to an increase in parasympathetic activity or a decrease in sympathetic activity [35]. These indirectly help in reducing the psychological parameters such as distress, anxiety, and depression in young healthy subjects.

There was significant reduction in LF power (*P* < 0.0002) whereas parasympathetic activity significantly increased (*P* < 0.0003). The LF powers indicate both sympathetic and parasympathetic modulation whereas HF power reflects parasympathetic activation. Hence reduction in sympathetic activity and enhanced parasympathetic activity results in deceleration in the cardiac activity. The ratio LF/HF, which is an indicator of autonomic ANS balance between sympathetic and parasympathetic nervous system activity [37], was significantly decreased (*P* < 0.0000). There is always constant interaction between sympathetic and parasympathetic activity to regulate heart rate variability. The VLF increased without significance in yoga group while there was significant increase in control group. The VLF represents slow changes in the heart rate [38], but the exact function of it is yet to be understood. A significant reduction in total power was observed in yoga group. This might be due to significant decrease of sympathetic activity compared to significant increase of parasympathetic activity. The VHF power generally reflects part of noise component and does not possess clinically significant information.

Control group showed significant increase of LF power (*P* < 0.0000), decreased HF power (*P* < 0.029), increased LF/HF ratio (*P* < 0.0000), and increased VLF power. But no significant changes were observed in time domain parameters.

The LF and HF band power of HRV are expressed in normalized units. The representation of these frequency band powers in normalized units articulates the degree of control exercised and the relative balance of two limbs of the autonomic nervous system [35]. Moreover normalized LF power is thought to represent the sympathetic modulation as opposed to absolute units. Since the HRV spectral parameters are computed by the autonomic nervous system (ANS), measurement of HRV may have greater application in assessing autonomic statues.

Student's paired *t*-test was performed on set of pre- and postintervention data samples to investigate whether there was any real difference between them. Each *t* value has a corresponding *P* value. The *P* value, which is the probability that the pattern of data samples in the sample could be produced by random data, provides the information about the likelihood that there is a real difference in the data pattern. This significant difference in the data set could be due to the effect of particular training or an intervention given to the subjects.

The various time and frequency domain parameters of both yoga and control group are shown in Table 5. Any variations in these parameters could be due to relative age differences between yoga and control groups or methodological differences or limited number of samples in the study.

The decrease in HR could be due to combined effect of elements of yoga. The reduction in stress after yoga could be other possible reason for improved HRV in this study. The previous researches suggest that yoga practice results in neurophysiological balance by lowering level of cholinesterase and catecholamines. Further, this result increased parasympathetic and decreased sympathetic activity. The results of this study are in concurrence with previous studies [6, 9, 35]. These studies indicated reduced sympathetic activity and enhanced parasympathetic activity after yoga.

### 3.2. Cognitive Performance Analysis

The regular practice of yoga for a period of five months by young healthy engineering students resulted in the increase of *α*, *β*, and *δ* EEG band powers and decrease in the *θ* and *γ* band powers. The increased *β* band power indicate enhancement in certain cognitive functions such as alertness, while increased *α* and decreased *δ* reflect enhanced vigilance level indicating increased alertness. Thus the increase of high frequency band powers (*α*, *β*) and decrease of low frequency band powers (*δ*, *θ*) are associated with enhancement in certain cognitive skills such as memory and visual information processing.

The various cognitive behavior parameters have been evaluated based on various EEG indices such as *θ*/*α*, *β*/*α*, *β*/*θ*, (*δ* + *θ*)/*α*, *β*/(*α* + *θ*), and (*δ* + *θ*)/(*α* + *β*). Increase in *β* band power indicates a higher level of alertness and enhanced engagement task and enhancement in various cognitive abilities. The increased band powers of *α* and *θ* indicate decreased alertness, reduced engagement task, and good information processing capabilities [25]. The *δ* and *λ* activity are used for analysis of many cognitive processes [39]. The ratio *β*/*θ* which is representative of improvement in cognitive skills increased. The heart rate index *θ*/*α* decreased, performance enhancement index *α*/*θ* increased, attention resource index *β*/(*α* + *θ*) significantly increased, executive load index (*δ* + *θ*)/*α* decreased, and ratio (*δ* + *θ*)/(*α* + *β*) decreased. The *α*, *β*, and *δ* band power increased in frontal, central, parietal, occipital, and temporal lobes. The *θ* band power was increased only in occipital lobe while *γ* band power in frontal and slightly in temporal lobes. As the frontal lobe is associated with reasoning, planning, problem solving, and cognition; parietal lobe with visual perception, recognition, information processing, and spatial reasoning; temporal lobe with memory and processing of verbal and auditory signals; and occipital lobe with visual spatial processing and recognition. An increase of EEG frequency band powers in these lobes indicates the enhancement of certain type of cognitive skills. The type of the cognitive skills developed can be assessed based on increased or decreased EEG band power in these lobes. The mean absolute values of these band powers in various lobes of the brain are shown in Table 6.

The increase of frontal *θ* band power indicates intellectual concentration and meditative state of relief from nervousness and is negatively related with sympathetic activation. This reflects a near relationship between autonomic function, activity of medial frontal neural circuitry, and probability of controlling central nervous system (CNS) functions owing to yoga practice and meditation [12]. *α* waves are indicative of increased relaxed state of mind and its band power is inversely related to mental activity. Yoga enhanced various cognitive skills, improved sense of wellbeing and responsiveness, and enhanced cognitive functions as substantiated by increased *α* and *β* band powers and various engagement indices. It also improves mental consciousness and achieves reduction in stress and strain and thus advocates complete health and wellbeing in an individual [40]. Increase in *β* band power would indicate a higher level of alertness and enhanced engagement task whereas increased band powers of *α* and *θ* would indicate decreased alertness and reduced engagement task [25]. The increase of *β* power reflects improvement of certain cognitive functions, such as memory and reaction time. That of *α* and *δ* indicates synchronization of brain activity. The total EEG band power also increased in yoga group compared to the control group.

The ratio *θ*/*α* is associated with HR. This ratio decreased in all lobes of the brain, indicating the relaxed state of subjects. This reduction could be either increase of *α* band power or decrease of *θ* band power. Since *α* power increased in yoga group this ratio decreased, indicating enhancement of certain cognitive faculties (memory, attention) and improvement in the HRV. These in turn indicate indirect improvement in certain cognitive functions such as reaction time. The ratio *β*/*α* [25] is called arousal index. This indicates level of arousal based on interbeat intervals (IBI) activity. Arousal level >0 indicates higher than normal arousal and <0 indicates lower than normal arousal.

This ratio increased (47.11%) in yoga group while decreased in control group (2.43%). The increases of this ratio indicate enhanced cognitive functions such as attention. The decreases of this ratio reflect reduction in the core capabilities of cognitive functions. This ratio increased in all lobes of the brain but the maximum increase was observed in parietal (78.05%), central (53.12%), and temporal (45.04%) lobes. It increased to (38.24%) and (19.76%) in frontal and occipital lobes, respectively. The ratio (*δ* + *θ*)/*α* is called executive load index [28] and is a measure of executive load or comprehension. Positive value indicates increased load and negative value indicates decreased load. It reflects the oscillations in precise cortical network active among spatially disjoint brain compartment. This ratio decreased (40.98%) in experimental group while it increased (17.19%) slightly in control group. The decrease in this ratio may be due to increase in *α* band power or reduced band power of either *δ* or *θ* or both. It is observed that *α* band power was increased in yoga practicing group. This ratio was also found decreased in all the lobes of the brain. Another important parameter *θ*(fro)/*α*(par) [27] is called task load index. This ratio decreases in experimental group while it increases in control group. It is evident from the relation that the ratio decreases due to increase in *α* band power. These results are shown in Tables 7 and 8.

The ratio *θ*/*β* indicates central nervous system (CNS) arousal [30] and increased ratio is marker of under arousal. This ratio decreased in yoga group. The reductions in this ratio reflect the shift of *α* and *θ* activity towards *β* activity. The increased *β* band powers indicate enhanced cognitive performance such as memory, attention, and concentration. The ratio *α*/*δ* which is called “brain perfusion” index [29] was increased (4.6%) in yoga practiced group. This indicates sufficient amount of blood flow to the different parts of the brain among yoga group. These in turn enhances the better functioning of the brain. The ratio LF/HF can be expressed in terms of (*θ* + *δ*)/(*α* + *β*) [29]. Slower brain oscillations (*θ* and *δ*) harmonize considerable neuron groups across larger brain areas and faster oscillations (*α* and *β*) synchronize smaller, focused neuronal assemblies [41]. When groups of neurons oscillate together synchronously, they more effectively communicated with each other. The index (*θ* + *δ*)/(*α* + *β*) indicates sympathovagal balance of the autonomic nervous system (ANS). Lower ratios indicate better balance of ANS. This ratio decreased in yoga group but did not change in control group. The ratio *α*/*β* [31] which is called desynchronization is used to analyze vigilance index and *δ*/*θ* is called synchronization. The ratio *α*/*β* decreased, which is desirable. The increase *β* of band power indicates improved cognitive performance. Since the parameter is in denominator, the ratio decreases when there is increased cognitive performance. However in control group this ratio was slightly increased. Another ratio *δ*/*θ* was increased relatively by small amount in experimental group than the control group. The *α*/*δ* ratio was decreased in frontal, central, and occipital lobes while it increased in parietal and temporal lobes. These results are shown in Tables 9, 10, and 11.

**Figure 1:**
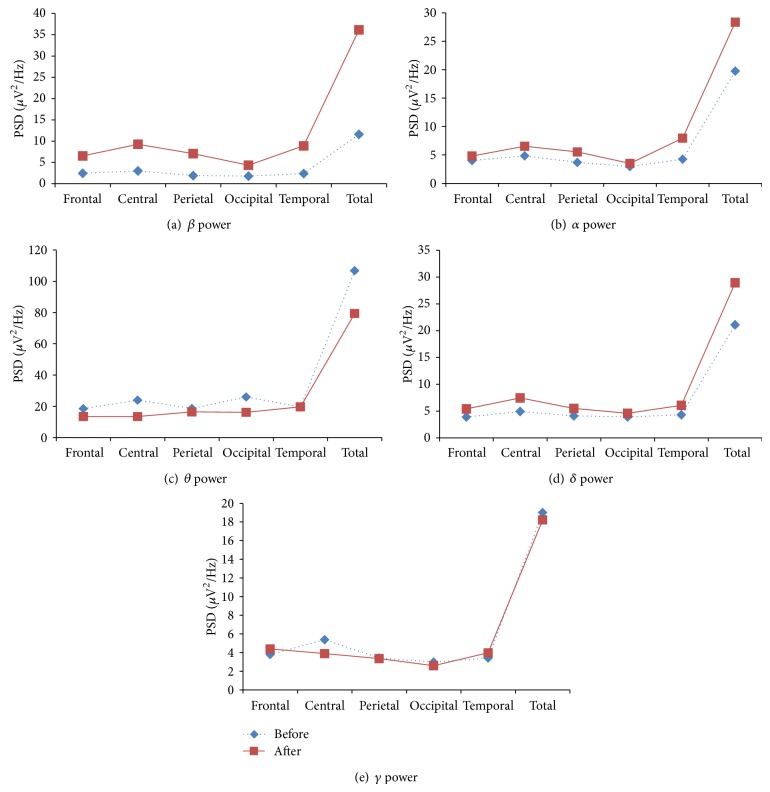
EEG band powers of yoga group in various lobes of the brain: before and after intervention.

The relative EEG band powers of *β*, *α*, and *δ* increased in yoga group in all the lobes of the brain. The increases of these band powers indicate improvement of certain cognitive functions such as memory, attention, executive functions, and concentration. The increase of *δ* power and decrease of *θ* power indicate improvement in neural activity. The various EEG band powers are shown in Figure 1.

**Figure 2:**
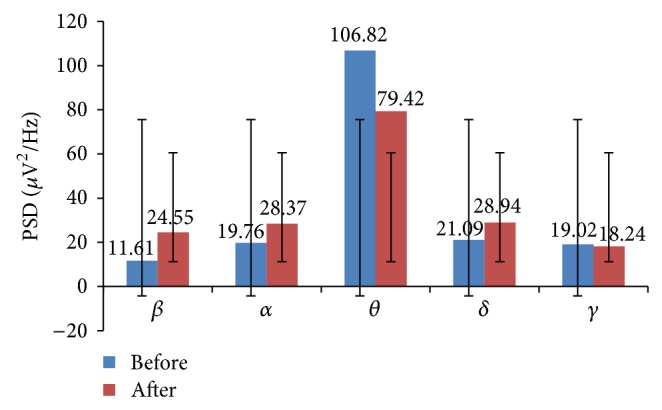
Global EEG band powers of yoga group: before and after intervention.

**Figure 3:**
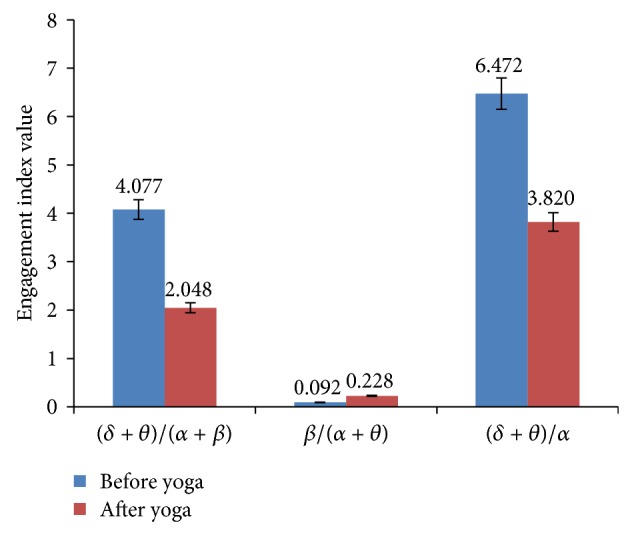
CNS activity, engagement index, and executive load index of yoga group: before and after intervention.

The total band power (global) of *β*, *α*, and *δ* increased among yoga group. The increased *β* power was associated with enhanced cognitive performance such as improved alertness. The maximum *β* power increased in frontal and central lobes of the brain. This indicates the improvement in emotion process and cognition. The increased *α* and decreased *θ* powers physiologically signify the enhanced vigilance and increased alertness level of subjects. These are shown in Figure 2. The improvement in cognitive functions was associated with increased power in high frequency band (*β*) and reduction in low frequency band (*θ*). Therefore *β*/*θ* ratio is suitable index to assess the improvement in cognitive skills of the subjects. The cognitive performance index *β*/(*α* + *θ*) increased and ratio of sum of low frequency to sum of high frequency (*θ* + *δ*)/(*β* + *α*) was decreased among yoga group, which is shown in Figure 3.

**Figure 4:**
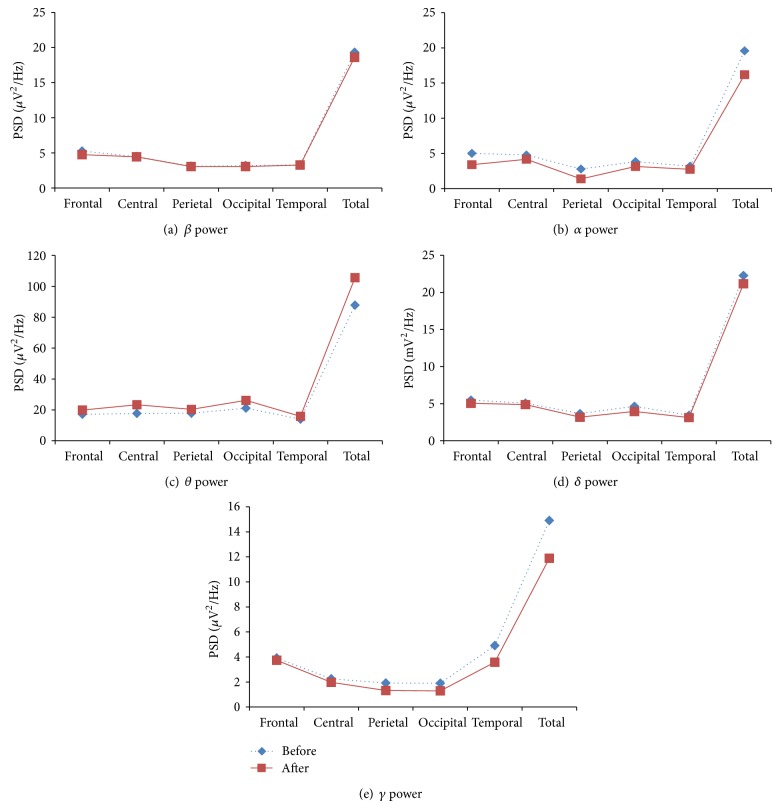
EEG band powers of control group in various lobes of the brain: before and after intervention.

**Figure 5:**
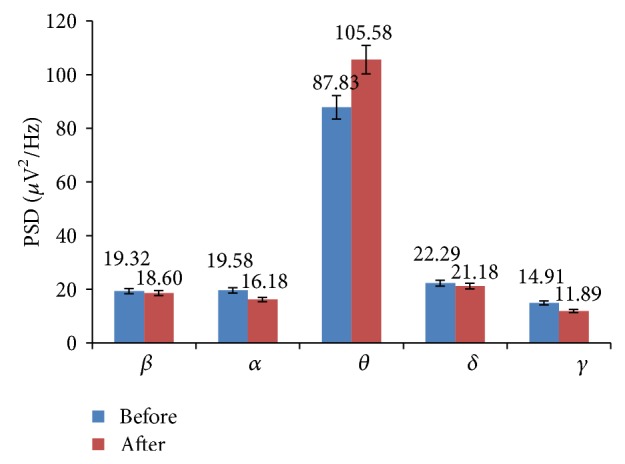
Global EEG band powers of control group: before and after intervention.

**Figure 6:**
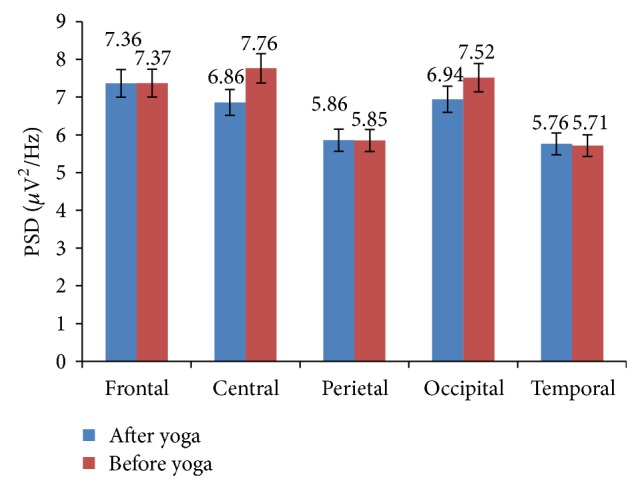
Global EEG band powers of control group in various lobes of the brain: before and after intervention.

**Figure 7:**
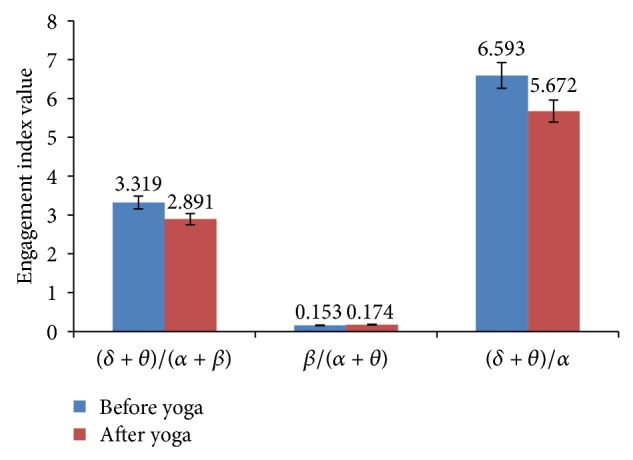
CNS activity, engagement index, and executive load index of control group: before and after intervention.

There were no significant changes in EEG band powers, engagement indices, total power, and cognitive performances indices among control group. The various performances parameters of control group are shown in Figures 4, 5, 6, and 7.

## 4. Conclusions

The regular practices of yoga for a period of five months by young healthy engineering students enhance different types of cognitive skills. Apart from cognitive, the yoga practice resulted in many health benefits such as improvement in heart rate variability. The ratio SDNN/RMSSD increased while the ratio LF/HF decreased. This indicates improvement in the parasympathetic activity and decrease in sympathetic activity. Hence the current results suggest that the practice of yoga modifies the sympathovagal balance towards parasympathetic activation, improved the heart rate variability, and enhanced sense of wellbeing. Since the study population is young healthy engineering graduates, it would be interesting to investigate whether the yoga practice could result in improvement in the academic performances.

In a nutshell, it is proved beyond doubt that yoga practices resulted in effective improvements in physiological parameters, indirectly improving psychological parameters and various cognitive functions. The results of this study greatly encourage further investigation to study whether the practice of yoga could also enhance academic performance.

## Figures and Tables

**Table 1:** The equations used to compute time domain measures.

Index	Equations	Unit
mHRV	$\frac{1}{N-1}\overset{N}{\underset{i=1}{\sum }}\left({\text{RR}}_{i}\right)$	ms

mHR	$\overset{N}{\underset{i=1}{\sum }}\left(\frac{1000}{{\text{RR}}_{i}}\right)*60$	bpm

SDNN	sqrt∑i=1NRRi-mRR2N-1∑sdsdsdsdsdt	ms

RMSSD	sqrtmean(RRi+1-RRi)2∑XXXtXXX	ms

CVRR	$\frac{\text{SDNN}}{\text{mean}\left(\text{RR}\right)}*100$	—

**Table 2:** The equations used to compute frequency domain measures.

Index	Equations	Unit
LF_n.u_	LFLF+HF∗100∑XXXXXXXXXX	%

HF_n.u_	HFLF+HF∗100∑XXXXXXXX	%

SVI	LFHF∑XXXXXXXX	—

LF_Rel.power_	LFTP∗100∑XXXXXX	—

HF_Rel.power_	HFTP∗100∑XXXXXXXXX	—

dLFHF	LFn.u-HFn.u∑XXXXXXXX	%

	where TP = VLF + LF + HF	

**Table 3:** Five EEG frequency bands.

Parameters	Frequency range (Hz)	Magnitude (*µ*V)	Activity	Remark
*β*	13–30	<30 *µ*V	Desynchronized	Mental occupation
*α*	8–13	50–100 *µ*V	Synchronized	Relaxed, tranquility, and wakefulness
*θ*	4–8	20–40 *µ*V	Desynchronized	Dreaming state
*δ*	0.5–4	75–150 *µ*V	Desynchronized	State of dreamless sleep
*γ*	30–70	—	Synchronized	Sensory integration

**Table 4:** EEG band ratios and their physiological/cognitive activity index interpretation.

EEG band ratios	Activity/correlation	Sources
*θ*/*α*	Heart rate (HR)	[23]
*α*/*θ*	Performance enhancement index or “wellbeing”	[24]
*β*/*α*	Arousal index	[25]
*β*/*θ*	Neural activity	[25]
*β*/(*α* + *θ*)	Cognitive performance and attentional resource index	[26]
*θ*(frontal)/*α*(parietal)	Task load index	[27]
(*δ* + *θ*)/*α*	Executive load index	[28]
*α*/*δ*	Brain perfusion	[29]
*θ*/*β*	CNS arousal	[30]
(*θ* + *δ*)/(*α* + *β*)	Sum of LF to HF ratio	[29]
*α*/*β*	Desynchronization	[31]
*δ*/*θ*	Synchronization	
(*θ* + *α*)/*β*	Vigilance index	[31]

**Table 5:** Time and frequency domain parameters, before and after intervention of yoga and control group.

Parameters	Yoga group			Control group		
	Before	After	*P* value	Before	After	*P* value
mHRV (ms)	757.21 ± 65.37	813.29 ± 78.91	0.0304	874.67 ± 91.55	855.72 ± 70.48	0.2505
mHR (bpm)	79.79 ± 7.74	74.57 ± 7.05	0.0389	69.50 ± 9.43	70.50 ± 6.22	0.2759
SDNN (ms)	44.43 ± 21.76	52.14 ± 23.27	0.0012	53.22 ± 21.69	53.83 ± 19.51	0.9044
RMSSD (ms)	39.93 ± 23.65	55.21 ± 22.78	0.0058	54.83 ± 29.46	49 ± 27.89	0.1999
SDNN/RMSSD	0.77 ± 0.37	1.11 ± 0.57	0.0039	0.77 ± 0.37	1.21 ± 0.28	0.1336
VLF (n.u)	4.02 ± 0.96	11.63 ± 9.83	0.0177	3.42 ± 9.91	14.04 ± 10.06	0.0007
LF (n.u)	123.06 ± 21.10	56.85 ± 10.16	0.0002	45.14 ± 13.09	41.57 ± 16.60	0.0000
HF (n.u)	27.39 ± 8.44	43.15 ± 10.16	0.0003	46.48 ± 4.61	2.20 ± 5.79	0.0269
LF_relative_	71.33 ± 5.67	51.16 ± 9.51	0.0000	46.42 ± 10.70	70.81 ± 7.13	0.0000
HF_relative_	27.39 ± 8.44	43.15 ± 10.16	0.0003	46.48 ± 4.61	42.20 ± 5.79	0.0269
TP (n.u)	171.99 ± 22.41	111.63 ± 9.83	0.0000	95.03 ± 11.29	97.81 ± 12.44	0.0000
LF : HF	4.99 ± 1.98	1.411 ± 0.45	0.0000	0.99 ± 0.32	3.55 ± 1.56	0.0000

*P* > 0.10 not significant; *P* < 0.10 marginal; *P* < 0.05 fair; *P* < 0.01 good; *P* < 0.001 excellent difference; *P* < 0.05 is considered significant level and for any value less than this; the null hypothesis is rejected. *t*_stat_ > *t*_valu_ for the null hypothesis to be rejected. If *P* = 0.05, there is 5% chance of no real difference.

**Table 6:** Mean powers of EEG frequency bands averaged across all the lobes of the brain before and after yoga intervention.

	EEG band powers				
	*γ* (*µ*V^2^/Hz)	*β* (*µ*V^2^/Hz)	*α* (*µ*V^2^/Hz)	*θ* (*µ*V^2^/Hz)	*δ* (*µ*V^2^/Hz)
Yoga group					
Before yoga	3.80 ± 0.93	2.32 ± 0.49	3.95 ± 0.70	21.36 ± 3.43	4.22 ± 0.42
After yoga	3.65 ± 0.69	4.91 ± 1.63	5.67 ± 1.68	15.88 ± 2.57	5.79 ± 1.06
Control group					
Before yoga	2.98 ± 1.36	3.86 ± 0.96	3.92 ± 0.97	17.57 ± 2.54	4.46 ± 0.89
After yoga	2.94 ± 1.33	3.72 ± 0.82	3.87 ± 0.90	21.12 ± 3.87	4.37 ± 0.78

**Table 7:** Cognitive index parameters of yoga group before and after yoga intervention. Postintervention values are shown within the parenthesis.

EEG indices	Yoga group				
	Frontal	Central	Parietal	Occipital	Temporal
*θ*/*α*	4.621 (2.806)	4.951 (2.063)	5.059 (2.973)	8.792 (4.609)	4.604 (2.480)
*α*/*θ*	0.216 (0.356)	0.202 (0.485)	0.198 (0.336)	0.114 (0.217)	0.217 (0.403)
*β*/*α*	0.612 (0.846)	0.625 (0.957)	0.523 (0.931)	0.607 (0.727)	0.564 (0.818)
*β*/*θ*	0.132 (0.302)	0.126 (0.464)	0.103 (0.313)	0.069 (0.158)	0.123 (0.330)
*β*/(*α* + *θ*)	0.109 (0.222)	0.105 (0.312)	0.086 (0.234)	0.062 (0.130)	0.101 (0.235)
*θ*(fro)/*α*(par)	4.621 (2.806)	4.951 (2.063)	5.059 (2.973)	8.792 (4.609)	4.604 (2.480)
(*δ* + *θ*)/*α*	5.590 (3.926)	5.964 (3.200)	6.168 (3.961)	10.112 (5.907)	5.614 (3.243)
∑(*δ* + *θ*)/*α* = 46.392 (27.876)					

**Table 8:** Global EEG band power ratios: before and after yoga intervention.

EEG indices	Yoga group		Control group	
	Before yoga	After yoga	Before yoga	After yoga
*θ*/*α*	5.405	2.800	4.485	5.460
*α*/*θ*	0.185	0.357	0.228	0.183
*β*/*α*	0.588	0.865	0.986	0.962
*β*/*θ*	0.109	0.309	0.220	0.176
*β*/(*α* + *θ*)	0.092	0.228	0.180	0.149
*θ*(fro)/*α*(par)	5.405	2.800	4.485	5.460
(*δ* + *θ*)/*α*	6.472	3.820	5.623	6.590
∑(*δ* + *θ*)/*α*	46.392	27.876	40.456	47.080

**Table 9:** EEG indices and their values: before and after yoga intervention.

Parameters	Yoga group		Control	
	Before	After	Before	After
*α*/*δ*	0.937	0.980	0.879	0.885
(*θ* + *δ*)/(*α* + *β*)	4.08	2.05	3.32	2.89
*α*/*β*	1.70	1.16	101.39	103.94
*δ*/*θ*	0.197	0.364	0.209	2.488
*θ*/*β*	9.198	3.235	5.530	4.721

**Table 10:** EEG index values of yoga group in different lobes of the brain: before and after yoga intervention. Postintervention values are shown in parenthesis.

Parameters	Brain lobes				
	Frontal	Central	Parietal	Occipital	Temporal
*α*/*δ*	1.032 (0.893)	0.988 (0.879)	0.902 (1.011)	0.757 (0.770)	0.990 (1.312)
(*θ* + *δ*)/(*α* + *β*)	3.468 (0.534)	3.669 (0.566)	4.050 (0.402)	6.294 (0.363)	3.589 (0.363)
*α*/*β*	1.634 (2.806)	1.599 (2.063)	1.913 (2.973)	1.648 (4.609)	1.772 (2.480)
*δ*/*θ*	0.210 (0.814)	0.205 (0.524)	0.219 (0.614)	0.150 (0.570)	0.219 (0.657)
*θ*/*β*	7.550 (3.316)	7.916 (2.156)	9.675 (3.194)	14.492 (6.341)	8.159 (3.032)

**Table 11:** EEG index values of control group in different lobes of the brain: before and after yoga intervention. Postintervention values are shown in parenthesis.

Parameters	Brain lobes				
	Frontal	Central	Parietal	Occipital	Temporal
*α*/*δ*	0.914 (0.944)	0.940 (0.940)	0.760 (0.760)	0.825 (0.825)	0.930 (0.931)
(*θ* + *δ*)/(*α* + *β*)	0.424 (0.356)	0.326 (0.261)	0.271 (0.241)	0.262 (0.218)	0.486 (0.438)
*α*/*β*	3.428 (4.186)	3.711 (4.891)	6.412 (7.311)	5.513 (6.817)	4.385 (4.970)
*δ*/*θ*	0.716 (0.741)	0.445 (0.445)	0.524 (0.523)	0.409 (0.409)	1.433 (1.433)
*θ*/*β*	3.269 (4.185)	3.958 (5.251)	5.747 (6.647)	6.633 (8.531)	4.235 (4.839)

## Limitations of the Study

Limitations of this study include small number of samples and lack of dedicated control group and methodological differences. Further investigation can be done by employing psychological tests to evaluate cognitive behavior.
